# Overview of Spiking Neural Network Learning Approaches and Their Computational Complexities

**DOI:** 10.3390/s23063037

**Published:** 2023-03-11

**Authors:** Paweł Pietrzak, Szymon Szczęsny, Damian Huderek, Łukasz Przyborowski

**Affiliations:** Institute of Computing Science, Faculty of Computing and Telecommunications, Poznan University of Technology, Piotrowo 3A Street, 61-138 Poznań, Poland

**Keywords:** spiking neural networks, learning algorithms, computational complexity, hardware

## Abstract

Spiking neural networks (SNNs) are subjects of a topic that is gaining more and more interest nowadays. They more closely resemble actual neural networks in the brain than their second-generation counterparts, artificial neural networks (ANNs). SNNs have the potential to be more energy efficient than ANNs on event-driven neuromorphic hardware. This can yield drastic maintenance cost reduction for neural network models, as the energy consumption would be much lower in comparison to regular deep learning models hosted in the cloud today. However, such hardware is still not yet widely available. On standard computer architectures consisting mainly of central processing units (CPUs) and graphics processing units (GPUs) ANNs, due to simpler models of neurons and simpler models of connections between neurons, have the upper hand in terms of execution speed. In general, they also win in terms of learning algorithms, as SNNs do not reach the same levels of performance as their second-generation counterparts in typical machine learning benchmark tasks, such as classification. In this paper, we review existing learning algorithms for spiking neural networks, divide them into categories by type, and assess their computational complexity.

## 1. Introduction

In the last decade, significant progress has been made in the field of neural networks. This progress mostly resides in the area of deep learning, which achieves high performance in fields like computer vision and natural language processing. Some notable tasks include object detection [1], image segmentation [2], text translation, and question answering [3]. SNNs however, are still not up to par with artificial neural networks in terms of the performance on common machine learning tasks. Classification datasets such as MNIST [4] and CIFAR-10 [5] still prove to be a challenge for these types of networks. Despite that, some of their applications have been developed by researchers. One such example is object detection. SNN achieved similar results to ANN while being much more energy efficient in terms of computations. A network was trained by using stochastic gradient descent and the KITTI dataset was used [6]. Another example from the domain of computer vision is image segmentation with UNET-based SNN. In this case, ANN was trained on the ISBI 2D EM dataset and converted to SNN [7]. Another application of machine learning using SNNs has been in LiDAR-based vehicles. The ability to autonomously control speed and steering in static and dynamic environments has been demonstrated [8].

An important trend in spiking neural network-based computer vision approaches are event-based cameras. These devices capture video with a fixed frame rate and only record changes in pixel intensity values. Their output is a series of on/off events that can be interpreted as input spikes for SNNs. They are called dynamic vision sensors (DVS). Their main benefits include low power consumption, high dynamic range, high temporal resolution, and lesser storage requirements [9]. In order to test spiking neural networks’ performance with DVS sensors, specialized datasets need to exist. Unfortunately, there are not many available, as DVS usually needs to be included during the process of dataset creation. Despite that, there have been some attempts at converting existing machine learning benchmark datasets to their event-based form. Examples include N-MNiST, N-Caltech101 [10], and CIFAR10-DVS [11].

SNNs are eagerly used in event-driven data analysis due to their high energy efficiency [12]. When considering SNNs in the context of energy efficiency, several application areas of SNNs should be mentioned. It was discovered that using SNNs in tasks related to reflective information processing can be efficient and can be applied in concept cells and the associated mathematical concept of a high-dimensional brain. Reflective SNNs can make use of their inherent dynamics to mimic complicated, nonreflexive brain functions, such as the creation of new skills from previously learned ones. SNNs can be implemented as analog computational systems [13]. In addition to the abovementioned areas of application, SNNs are also used in the areas of cognitive processing. One application is a model of spatial memory implemented in an SNN that was used on a robot moving in an environment with neutral and harmful regions. In that application, STDP rearranges the couplings in the SNN and forms spatial memory similar to cognitive maps associated with the negative experience that resulted in a learning robot to avoid harmful zones [14]. Another area of cognitive development is the application of SNN for associative learning of perceptual information. That approach refines the relationship among the perceptual information and can reflect the relationship to the natural communication with a human. The proposed method was applied in Partner Robot which learns through interaction with people [15].

Robots typically have limited hardware resources, and thus energy-efficient SNNs running on event-based asynchronous hardware can be a perfect fit for these use cases. There have been some applications of SNNs here as well. An SNN running on Loihi was used to solve unidimensional simultaneous localization and mapping (SLAM) problem and achieved comparable accuracy to the GMapping algorithm while being 100 times less energy consuming [16]. Another great example is the use of reward modulated spike timing-dependent plasticity (R-STDP) algorithm to train an SNN to control a robotic arm [17]. A lot of research is still going on into the use of SNNs in hardware implementations, especially in chips for use in robotics and autonomous intelligent systems [18]. Furthermore, the SNN architecture has been shown to solve an unsolved problem in classical control theory for telerobotics [19]. An interesting solution is the implementation of neural networks for keyword spotting and adaptive robotic control on the prototype chip of the SpiNNaker 2 neuromorphic system. Keyword spotting is commonly used in smart speakers to listen for wake words, and adaptive control is used in robotic applications to adapt to unknown dynamics online. The SpiNNaker 2 prototype uses a multiply–accumulate (MAC) array, which is typically used in pointer-based machine learning networks when applied in a neuromorphic context [20]. Another interesting application of spiking neuron models is the representation of the motor cortex and cerebellum of the motor control system using them. The designed model consists of anatomically organised spiking neurons covering the premotor, primary motor, and cerebellar cortices. New neural computations in these areas were obtained to control a nonlinear, trilinear model of the arm that can adapt to unknown changes in the dynamics and kinematic structure of the arm. The mathematical stability of both forms of adaptation is demonstrated, suggesting that this is a robust approach to the common biological problems of body size change and unexpected dynamic perturbations [21]. The SNN has also been implemented with a quadrodron algorithm that has a total delay of 3.5 milliseconds, which is sufficient to reliably detect and avoid fast-moving obstacles [22]. A number of neural network simulation tools have been developed. One such tool is the Nengo library set. It enables the building and development of SNNs running on CPU and GPU and Intel’s Loihi neuromorphic chip [23].

Spiking neural networks closely mimic the human brain. They use discrete events called spikes in opposition to artificial neural networks by using scalar values. Due to this fact, a single response of an SNN needs to be computed over several time steps (the number may vary by individual architectures and learning algorithms). This fact causes them to be less efficient on standard synchronous computer hardware than ANNs because of the need to query the model in a time-step loop. However, these networks may be much more efficient on specialised neuromorphic hardware. Such hardware is made of asynchronous circuits and is event driven [24]. Hardware asynchronicity allows for sparsity of data streams in SNNs. It is known that less than 10% of neurons in the brain are simultaneously active [25]. This property is not utilized in ANNs, as all neurons are active in every forward pass of the network. Data sparsity also raises efficiency of local learning rules for neural networks. Local learning rules only modify parts of the network based on the activity of subset of neurons. A prime example of a learning algorithm in this category is spike timing-dependent plasticity (STDP) and its derivatives [26]. An opposite learning approach that involves all neurons in every iteration is backpropagation. This approach is used for ANNs, and it involves the use of loss function that describes how good/bad the network performs a given task. It minimizes the loss through updating the weights based on their individual gradients [27]. The importance of the software–hardware ecosystem is crucial for the further development of neurorobotics. One of the most important frameworks for this is the neural engineering framework (NEF), which is described in detail in an extensive body of work [28]. More about this framework, and also about neuromorphic programming, neuromorphic architectures, hardware, and circuits can be found in [29]. The topic of mapping the AI model to neuromorphic hardware by using NEF was discussed in the work [30].

Currently existing neuromorphic architectures include

- IBM TrueNorth,
- Intel Loihi,
- Tianjic,
- SpiNNaker,
- BrainScaleS,
- NeuronFlow,
- DYNAP, and
- Akida.

Some of the above architectures are fully neuromorphic [31,32], while other remain hybrid, meaning that they use asynchronous circuits together with synchronous processors [33,34]. Despite the field being still in its infancy, the first commercial neuromorphic processor was made available worldwide in August 2021. It is Akida from Australian company BrainChip. Unfortunately, these hardware platforms are very expensive at the time of writing and (apart from Akida) not feasibly available.

A more widely available alternative to neuromorphic hardware is the category of synchronous digital computer hardware. Most notable examples include:

- CPUs
- GPUs
- TPUs (Tensor Processing Units)
- FPGAs (Field Programmable Gate Arrays)
- VPUs (Vision Processing Units)

All of the above hardware platforms are characterized by synchronicity of events (synchronization using a clock signal in the circuits) and some degree of operation parallelization. For CPUs and GPUs, this parallelization lies in the multiplicity of cores that perform operations. CPUs typically have high clock rates and low numbers of cores (anywhere from 1 to 32), whereas GPUs have low clock rates and large numbers of cores (ranging even up to couple thousand). Due to the massive parallelization of execution, GPUs are much more efficient for deep learning with ANNs than CPUs. TPUs fall in the category of application-specific integrated circuit (ASIC) AI accelerators. They were developed by Google and are used mostly for deep learning acceleration. FPGAs, however, differ from the other positions on the list because they contain reconfigurable hardware that allows one to emulate various digital electronic circuits, e.g., CPUs (emulated CPUs in this case are called soft processors). FPGAs are very flexible in terms of reconfiguration, but the limiting factors here are available hardware resources. For example, their usage in deep learning is severely limited due to the scarcity of hardware multipliers in comparison to GPUs and TPUs. These platforms, however, can be beneficial for implementations of small, sparsely connected networks, which is often the case with SNNs. Configurable custom circuits on an FPGA can guarantee implementation of such networks with minimal hardware resources used and that can be advantageous for putting together complex AI-driven systems on a chip (SoCs). VPU is a type of AI accelerator that focuses on computer vision domain. That translates mostly to the acceleration of convolution-based machine learning algorithms, e.g., convolutional neural networks.

As mentioned, the subject of SNNs involves many different hardware architectures and many different applications and research areas. SNNs are also the subject of many review articles [13,35]. In this review, we focus on the main learning approaches for SNNs in terms of their efficiency on synchronous digital hardware. Out of all synchronous digital hardware platforms, CPUs and GPUs are the most widely used; thus we narrowed down our analyses to these two platforms. We collected the key parameters of the learning algorithms and assessed the complexity of the algorithms. We hope that this work will be useful mainly for researchers considering the use of neurocomputation in the analysis of data from various types of sensors [36,37,38]. In chapter two, we will briefly discuss the most common neuron models, synapse models, and input encoding types. In chapter three, we will provide an overview of existing learning algorithms for SNNs. In chapter four, we will analyze these learning algorithms in terms of their computational complexities. Finally, in chapter five, we will provide a summary of the current state of SNNs and draw conclusions from that.

## 2. Spiking Neural Networks Fundamentals

### 2.1. Neuron Models

**Figure 1:**
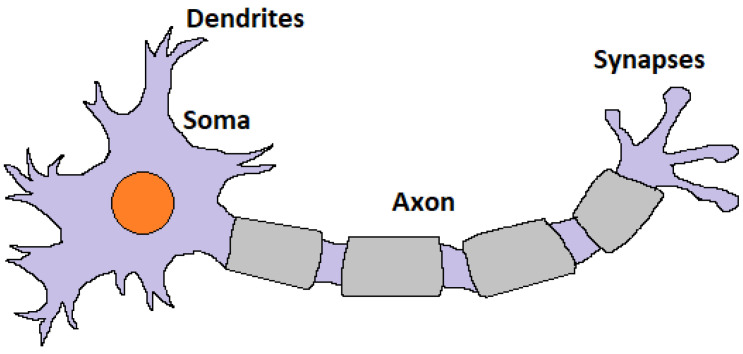
Biological neuron’s structure.

Neurons in the brain propagate electrochemical signals through action potentials. Both in neurons and extracellular fluid, ions exist and flow constantly in and out of cell membranes. This electrochemical signalling causes current flow. Neurons consist of four parts: synapses, dendrites, somas, and axons. Synapses form connections between neurons and they reside on dendrites, which are short nervous connections that handle direct input to neurons. Their main task is translation of chemical signals into electric signals. Soma is the cell body. All membrane potentials from synaptic inputs are integrated there. Integration process determines whether the postsynaptic cell fires the action potential. The axon carries action potentials to other cell’s synapses [35]. A neurons’s structure is shown in Figure 1.

There have been various attempts at describing biological neurons’ functionality as a computational model. In the following sections, we will describe most popular ones. The variables used in the models are listed in Appendix A at the end of the article.

#### 2.1.1. Integrate and Fire

The most computationally efficient model, as well as the least biologically plausible, is Integrate and Fire (IF). Equation (1) portrays I&F neuron’s mechanics. $\frac{dV}{dt}$ is the derivative of neuron’s potential over time and I(t) is the total current from synapses. When a neuron’s potential reaches a certain threshold, a spike is fired [39]. We have

(1) $$C_{m}\frac{dV}{dt}=I\left(t\right).$$

#### 2.1.2. Leaky Integrate and Fire

A Leaky Integrate and Fire (LIF) neuron is a version of Integrate and Fire with a potential leakage. The leakage is represented by $-g_{L}\left(V-E_{L}\right)$ part of the Equation (2). $E_{L}$ is the resting potential, *V* is the previous membrane potential, and $g_{L}$ is the leak conductance. Leakage models the diffusion of ions that occurs through the membrane when an equilibrium is not reached in the cell [35,40]. We have

(2) $$C_{m}\frac{dV}{dt}=I\left(t\right)-g_{L}\left(V-E_{L}\right).$$

#### 2.1.3. Izhikevich

A step further in terms of biological plausibility is an Izhikevich neuron model. This model retains relatively low computational complexity, while maintaining a broad range of possibilities in terms of neuron behaviour. Examples of such behaviours include regular spiking, bursting, chattering, and fast spiking. Equations (3)–(5) describe this neuron’s mechanics. *u* is the recovery variable, and *a*, *b*, *c*, *d* are constants, the different values of which allow for different spiking behaviours [41]. We have

(3) $$\frac{dv}{dt}=0.04v^{2}+5v+140-u+I$$

(4) $$\frac{du}{dt}=a\left(bv-u\right)$$

(5) $$if\;\;v\geq 30\;\mathrm{mV},\;\;then\left\{\begin{array}{c} v\leftarrow c\\ u\leftarrow u+d \end{array}\right..$$

#### 2.1.4. Hodgkin–Huxley

The Hodgkin–Huxley neuron model was developed after conducting an experiment on the axon of a squid. It takes into consideration three ion channels: $NA^{+}$, $K^{+}$, and $CI^{-}$. These independent channels are described in Equation (6), and overall neuron mechanics are described in Equation (7). This neuron model is biologically accurate; however, it is computationally expensive, and thus it is infeasible for large simulations [35]. We have

(6) $$I_{ion}\left(t\right)=G_{K}n^{4}\left(v_{m}-E_{K}\right)+G_{Na}m^{3}h\left(v_{m}-E_{Na}\right)+G_{L}\left(v_{m}-E_{L}\right)$$

(7) $$\frac{dV}{dt}=I_{ion}\left(t\right)+I_{syn}\left(t\right).$$

### 2.2. Synapse Models

Synapses produce current in the presence of voltage spikes coming from other neurons. There are many synapse models, and they differ in terms of biological plausibility as well as computational efficiency. In this subchapter, we will review the two most popular models that are used in simulations and are considered to be relatively efficient: current-based synapse models and conductance-based synapse models.

#### 2.2.1. Current-Based Synapse Model

The current-based synapse model is described by Equation (8). *g* is the synapses conductance that corresponds to its weight, $v_{sp}$ is spike voltage level, and *n* is the number of input synapses to a neuron. Input currents are summed by using Kirchhoff’s first law. This synapse is widely used in many applications due to its simplicity [17,42,43]. We have

(8) $$I\left(t\right)=\Sigma_{i=1}^{n}g_{i}v_{sp}.$$

#### 2.2.2. Conductance-Based Synapse Model

The conductance-based synapse model is similar to the current-based synapse model, but here equilibrium synapse potential $E_{eq}$ and additional conductance mechanics are introduced. In the presence of spikes, conductance is increased by the amount of weight corresponding to the given synapse. In the absence of spikes, conductance decays exponentially [26]. These mechanics are shown in Equations (9) and (10). $\tau_{g_{e}}$ in Equation (10) is the time constant of an excitatory postsynaptic potential. We have

(9) $$I\left(t\right)=\Sigma_{i=1}^{n}g_{i}\left(E_{eq}-v_{sp}\right)$$

(10) $$\tau_{g_{e}}\frac{dg}{dt}=-g.$$

### 2.3. Encoding Types

Due to the temporal domain of spikes in SNNs, all inputs must be encoded as spikes, which retain relations between all data points. Two main encoding schemes exist: rate encoding and temporal encoding.

#### 2.3.1. Rate Encoding

Rate encoding scheme is based on the average number of spikes over a designated time interval. In the simplest case, all spikes can be spaced evenly across the time axis. A more sophisticated and biologically plausible approach is usually preferred: generating spike events as a Poisson process with a constant average firing rate [26,35]. This encoding method implies that only average firing rates are important for inference, which is not ideal but is effective in practice and can be implemented in hardware [44].

#### 2.3.2. Temporal Encoding

Temporal encoding scheme is based on the individual timing of spikes. Most of the time in this encoding scheme, one-spike-per-neuron networks are used. In this case, the delay of the spike is inversely proportional to the corresponding input value. An example here would be a pixel value 55 from <0–255> range and a 512 ms time interval. This specific pixel would be encoded with a time of $\frac{(256-55)*512}{256}=402$ ms [35,42]. Temporal encoding is very efficient and maps well to hardware [44].

## 3. Types of Learning Approaches

### 3.1. STDP

Spike timing-dependent plasticity (STDP) is an unsupervised learning algorithm that is biologically plausible [45] and is based on a hebbian rule that can be summarised as follows: if two connected neurons fire at the same time, the weight of the synapse between them should be strengthened [46]. In STDP, however, if a presynaptic spike precedes a postsynaptic spike, the weight of the synapse is either strengthened or weakened and similarly, in the opposite scenario, when a postsynaptic spike precedes a presynaptic spike the weight of the synapse is either weakened or strengthened. If in the first situation weight is strengthened and in the second weakened, STDP is called hebbian STDP. In the reverse setup, STDP is called anti-Hebbian STDP.

The function that governs how much the weight of the synapse is changed is called a learning window. An example of a popular hebbian STDP window is presented in Equation (11) and antihebbian version is shown in Equation (12). There are many types of learning windows for STDP [26]. We have

(11) $$\Delta =\left\{\begin{array}{c} A_{+}exp\left(\frac{t_{pre-t_{post}}}{\tau_{+}}\right)\;if\;t_{pre}\leq t_{post}\\ A_{-}exp\left(-\frac{t_{pre-t_{post}}}{\tau_{-}}\right)\;if\;t_{pre}>t_{post} \end{array}\right.$$

(12) $$\Delta =\left\{\begin{array}{c} A_{+}exp\left(\frac{t_{pre-t_{post}}}{\tau_{+}}\right)\;if\;t_{pre}\geq t_{post}\\ A_{-}exp\left(-\frac{t_{pre-t_{post}}}{\tau_{-}}\right)\;if\;t_{pre}<t_{post} \end{array}\right..$$

In the above equations, $A_{+}$ and $A_{-}$ are scaling factors for potentiation and depression of the synapse, $t_{pre}$ and $t_{post}$ are presynaptic and postsynaptic spike times, and $\tau_{+}$ and $\tau_{-}$ are time constants of synapse potentiation and depression.

There are other types of STDP. An example would be reward-modulated STDP [17,35], which is an STDP mechanism combined with reward signal. The reward signal governs whether synapses get potentiated or depressed. This mechanism is based on dopaminergic neurons in the brain [47]. Other, less popular, variants of STDP include mirrored STDP [48] and probabilistic STDP [49]. Mirrored STDP is an attempt to implement autoencoders in a biologically inspired manner. It combines STDP and antihebbian STDP for feedforward and feedback connections in a two-layer autoencoder-type network. Probabilistic STDP adjusts the synaptic weight according to an exponential function of the current weight magnitude (Equation (13)). $\eta_{+}$ and $\eta_{-}$ are potentiation and depression learning rates, and w is the synaptic weight being adjusted. We have

(13) $$\Delta w=\left\{\begin{array}{c} \eta_{+}exp\left(-w\right)\;if\;t_{pre}\leq t_{post}\\ -\eta_{-}\;if\;t_{pre}>t_{post} \end{array}\right..$$

Most of the time, the key to a successful application of STDP lies in balancing stability of the algorithm and neuron competition. The first case can be addressed by modifications of the learning window, i.e., multiplying by a factor dependent on a maximum weight value allowed: $\left(w_{max}-w\right)$. Other approaches involve applying homoeostatic mechanisms to neurons. An example of such mechanism would be increasing the spiking threshold whenever a neuron spikes and decreasing it exponentially until an initial value is reached in the absence of spikes [26].

Enhancing the competition of neurons can be done by introducing lateral inhibition mechanisms. These mechanisms allow neurons that spike first to inhibit responses of neighbouring neurons. In the brain, lateral inhibition usually leads to winner-take-all (WTA) situations between neurons [50]. However, in the case of using STDP to solve practical problems, various degrees of inhibition need to be introduced in consecutive layers. In the case of classification layer, high lateral inhibition (WTA behaviour) is desired, whereas in the former layers the WTA mechanism may lead to the loss of information during the forward pass of the network. A bioinspired way by which to introduce lateral inhibition to neurons is to connect an additional inhibition layer that allows each neuron to inhibit its neighbours via backward connections [26]. Some analytical approaches for implementing lateral inhibition mechanism were developed as well [51,52].

### 3.2. Backpropagation

Backpropagation-based approaches do not aim for biological plausibility, as is the case for STDP. Instead, they focus on learning complex spatiotemporal relations between spikes. This comes with a severe drawback of long training times, as networks need to conduct forward passes frequently, which on standard computer hardware takes a long time even with parallelization. This is due to the necessity of querying the network over several time steps to produce one spike reponse per input. Temporal encoding and one-spike-per-neuron networks can minimize this problem somewhat, but we will review this approach in the next chapter.

Backpropagation cannot be used directly on SNNs due to the problem of nondifferentiable neuron equation. Because of this, the derivative needs to be approximated in order for backpropagation to work. These approximations can be made around spiking time [53], membrane potential [54], ReLU activation function [55], or even the STDP mechanism [42].

Spikeprop is the first event-based backpropagation method invented for SNNs. In the original conference paper, it was used to solve the XOR problem with a feedforward network of two input neurons, four hidden neurons, and one output neuron [53]. It uses least mean squares error as the loss function,

(14) $$E=\frac{1}{2}\Sigma_{j}{\left(t_{j}-t_{j}^{d}\right)}^{2},$$

where $t_{j}$ is the spike time, and $t_{j}^{d}$ is the desired spike time. The nondifferentiability problem is alleviated here by substituting spike time $t_{j}$ with a linear function of t. This function, called threshold function, is defined as $\delta t_{j}=-\delta x_{j}\left(t_{j}/\alpha \right)$, where $x_{j}$ is the post-synaptic input of *j*th neuron and α is the local derivative of $x_{j}$ with respect to t: $\frac{\delta x_{j}\left(t\right)}{\delta t}$ when $t=t_{j}$ [53].

Superspike is an improved version of the Spikeprop algorithm, as the derivative approximation is based on membrane potential instead of spike times. This allows the network to be trained in the absence of spikes, which renders the learning process immune to the “dead neuron” problem. Input to the trained network is a Poisson spike train and the output of the network is a spike train with a certain frequency. The loss function chosen was the van Rossum distance between the output and desired spike trains together with sum-squared error. The Superspike loss function is shown in Equation (15) [54],

(15) $$L=\frac{1}{2}\int_{0}^{T}{\left(\alpha *\left(s\left(t\right)-s^{\prime }\left(t\right)\right)\right)}^{2}dt,$$

α is a normalised smooth temporal convolution kernel, *s* is the output spike train, and $s^{\prime }$ is a target spike train. During the calculation of the derivative with respect to the weights, the term containing the Dirac delta function appears, which is nondifferentiable. To avoid this term, spike trains are approximated by using a continuous function of the membrane potential of the LIF model. The approximation is shown in Equation (16). σ(x) represents a fast sigmoid function, $v_{m}$ is the membrane potential, and *w* is the weight [54]. We have

(16) $$\frac{\delta s}{\delta w}=\frac{\delta \sigma (v_{m})}{\delta w}=\sigma^{\prime }\left(v_{m}\right)\frac{\delta v_{m}}{\delta w}.$$

The supervised spike timing-dependent plasticity (SSTDP) is an event-based backpropagation approach that can train low-latency networks, with simulation times as low as 16 time steps. This method is based on one-spike-per-neuron networks consisting of IF neurons and temporally encoded inputs. Loss calculation is based only on single spike timings, which makes the training process much faster than other methods, but predisposes the algorithm to the “dead neuron” problem [42]. Loss function is shown in Equation (17). We have

(17) $$E=\frac{1}{2}\Sigma_{j=1}{\left(t_{j}^{L}-T_{j}\right)}^{2},$$

where $T_{j}$ is the expected firing time that is calculated adaptively based on the network’s response on current input sample. Its formula is shown in Equation (18),

(18) $$T_{j}^{L}=\left\{\begin{array}{c} min\;t_{j}^{L},T_{mean}-\frac{n-1}{n}g,\;j=y\\ max\;t_{j}^{L},T_{mean}+\frac{1}{n}g,\;j\neq y \end{array}\right.,$$

where *n* is the number of output spikes, *y* is the correct label, and $t_{j}^{L}$ is the actual firing time. This setting maintains the average expected firing time near the actual average firing time to achieve better adaptation during learning. Regarding the problem of nondifferentiable terms, the derivative of loss over weights is calculated as a nondifferentiable expression: $\frac{\delta t^{l}}{\delta V^{l}}\frac{\delta V^{l}}{\delta w^{l}}$. The authors decided to use a different approach from previous works and merge these terms into one: $\frac{\delta t^{l}}{\delta w^{l}}$, which is calculated by using STDP [42]. We have

(19) $$\frac{\delta t_{j}^{l}}{\delta w_{ij}}=\left\{\begin{array}{c} \epsilon_{1}\left(e^{\frac{t_{post}-t_{pre}}{\tau }}\right){\left(w_{max}-w\right)}^{\mu },\;t_{post>t_{pre}}\\ \epsilon_{2}\left(e^{\frac{t_{post}-t_{pre}}{\tau }}\right){\left(w_{max}-w\right)}^{\mu },\;t_{post<t_{pre}} \end{array}\right..$$

The SLAYER algorithm takes a different approach than event-based methods as it distributes the credit of error back in time. This solves issues with previously mentioned approaches, that credit the error in given time steps only and neglect the effect of earlier spikes [56]. Equations (20) and (21) depict SLAYER’s loss function mechanics. We have

(20) $$L=\frac{1}{2}\int_{0}^{T}{\left(e^{n_{l}}\left(s^{n_{l}}\left(t\right)\right),s^{\prime n_{l}}\left(t\right)\right)}^{2}dt$$

(21) $$e^{n_{l}}\left(t\right)=\epsilon *\left(s^{n_{l}}\left(t\right)\right)-s^{\prime n_{l}}\left(t\right)),$$

where *s* is the output spike train and $s^{\prime }$ is a target spike train. ϵ is the spike response kernel which distributes the effect of input spikes into the future time values. The problem of derivative approximation is solved by the usage of probability density function, which represents the likelihood of neurons changing state from nonspiking to spiking [56]. SLAYER is similar to Superspike in regard to inputs and outputs to the network, as inputs are Poisson spike trains and outputs are spike trains with a certain frequency.

The last backpropagation approach that we have chosen to discuss allows learning synaptic weights together with membrane time constants and was presented in [57]. This approach is based on a special Parametric Leaky Integrate and Fire (PLIF) neuron that also follows the mechanics of the standard LIF neuron (Equation (2), where the membrane time constant is $\tau_{m}=C_{m}/g_{L}$), but has three modifications:

(1) Membrane time constant $\tau_{m}$ is optimized during training and isn’t set as a hyperparameter.
(2) Membrane time constant $\tau_{m}$ is shared within all neurons in the same layer.
(3) Membrane time constants $\tau_{m}$ are distinct across all layers.

Tested SNN consists of two parts: convolutional and fully connected. The unique aspect of this approach is that the convolutional part performs input encoding, so the inputs are not converted to spikes beforehand. Output encoding is rate based. Despite the rate encoding, authors were able to achieve competitive results with very limited time steps [57]. Authors also use their own implementation of max pooling in the network.

The loss function used for training is mean squared error (MSE):

(22) $$L=\frac{1}{T}\Sigma_{t=0}^{T-1}\frac{1}{C}\Sigma_{i=0}^{C-1}{\left(o_{t,i}-y_{t,1}\right)}^{2},$$

where *T* is the number of simulation steps, *C* is the number of classes equal to the number of neurons in the output layer, $o_{t,i}$ is an output tensor of shape C × T, and $y_{t,i}$ is the label of same shape as output but with 1s in places corresponding to the neuron of the desired class and 0s elsewhere. This is done to maximize the activity of the desired neuron because class prediction is determined on an output neuron with maximum firing activity (total number of spikes in T). The problem of nondifferentiability comes here in the partial derivative of spike over membrane potential: $\frac{\delta S_{t}^{i}}{\delta H_{t}^{i}}$, which is solved by using a derivative of surrogate function σ:

(23) $$\sigma \left(x\right)=\frac{1}{\pi }arctan\left(\pi x\right)+\frac{1}{2}$$

(24) $$\sigma^{\prime }\left(x\right)=\frac{1}{1+{\left(\pi x\right)}^{2}}.$$

### 3.3. ANN–SNN Conversion

In terms of SNN learning algorithms, conversion from deep learning models to SNNs usually achieves best results on common supervised-learning benchmark datasets, such as MNiST, CIFAR10, and Imagenet [35,43]. In this approach, an ANN is trained by using backpropagation and converted to SNN by adapting weights and parameters of the spiking neurons. The goal is to achieve the same input–output mapping as in the ANN. Conversion approaches can be divided into two groups: regular conversion and contrain-then-train conversion. The main difference between the two is that in the first category of approaches ANN is trained once and can be converted multiple times to SNNs of different parameters (neuron types, time constants, reset voltages, etc.) and in the second category ANN is constrained during training to ease the conversion process towards a designated neuromorphic hardware and SNN architecture; thus the ANN training needs to be repeated if the change happens in the architecture of the network [58].

Conversion approaches focus mostly on computer vision domain and CNN-to-SNN conversion [43]; however, there has been an attempt at converting recurrent neural networks as well [59]. Resulting SNNs usually are rate-coded, but there have been attempts to use latency coding as well [60]. Most conversion approaches are based on converting neurons with rectified linear unit (ReLU) activation function to IF neurons. The reason behind the use of ReLU is that it is equivalent to IF neurons in the spiking domain without leak and refractory period [43]. The main idea behind ANN-SNN conversion is to map the analog value of activation function to firing frequency or average postsynaptic potential of a spiking neuron. Equation 25 presents calculations done in a single layer of neurons in the ANN and Equation (26) shows ReLU activation function. We have

(25) $$a^{l}=h\left(W^{l}a^{l-1}\right),\;l=1,2,...,M$$

(26) h=max(0,x),

where $W^{l}$ is the weight matrix, $a^{l}$ and $a^{l-1}$ are outputs of subsequent layers, and h is the ReLU activation. Equation (27) presents the relationship of the average postsynaptic potential of spiking neurons in subsequent layers [61],

(27) $$\phi^{l}\left(T\right)=W^{l}\phi^{l-1}\left(T\right)-\frac{v^{l}\left(T\right)-v^{l}\left(0\right)}{T},$$

where $\phi^{l}$ and $\phi^{l-1}$ denote average postsynaptic potential before time T, $W^{l}$ is the weight matrix between layers l and l − 1, and $v^{l}$ is neuron’s potential after firing. When initial potential $v^{l}\left(0\right)$ is equal to 0 and simulation time T is long enough, Equation (27) is equivalent to Equation (25) with ReLU activation [61]. In general, long simulation times minimize the decrease of SNN’s performance in comparison to the original ANN but drastically increase the output network’s latency, whereas short simulation times increase prediction error but grant shorter response latency [43]. A direction of research studying other encoding types in ANN-SNN conversion aims to mitigate the problem of long simulation times [60].

ANN-SNN conversion has other flaws. One drawback is that ANN’s activation cannot be negative, because then it cannot be represented in spikes. This is also the main reason why ReLU is mostly used in this SNN learning technique [35]. Another limitation, which is connected specifically to CNN-SNN conversion, is difficulty in implementing max pooling operation in the spiking domain due to its nonlinearity. Most works opted to replace this operation with average pooling in the ANN [43,62], but it decreases the network’s performance. A simple mechanism for implementation of max pooling in the spiking domain is presented in [63], which uses gating functions that pass spikes from only maximally firing neurons.

### 3.4. Comparison of Benchmarking Results

Table 1 presents comparison of highest classification accuracy per learning approach on MNiST and CIFAR-10 datasets.

As we can see, the highest results are achieved by backpropagation and ANN-SNN conversion approaches [57,64,65]. In the backpropagation row in the table, both state-of-the-art results were set by the method described as last in Section 3.2 [57].

## 4. Computational Complexity Analysis

The main problem for SNNs in terms of computational complexity is the network’s simulation time. The need to query SNNs over several time steps to achieve one forward pass makes them inferior to ANNs on standard computer hardware. Their sparsity of computation, however, can make them more energy efficient on neuromorphic hardware. Unfortunately, this hardware is not widely available, and neuromorphic architectures differ from each other in terms of SNN’s architectural requirements. In this chapter, we will review the computational complexities of all the main learning approaches on standard computer hardware.

### 4.1. STDP

STDP can be implemented in two ways: by a standard or online version. In the standard version, weight changes are computed after all forward passes have been made by using presynaptic and postsynaptic spikes and the STDP learning window. This version, depending on how many presynaptic spikes and postsynaptic spikes correlate to cause weight changes, can be extremely memory intensive and computationally heavy. In order to compute weight changes after forward passes, presynaptic and postsynaptic spike histories need to be stored in memory. They can be stored as two one-dimensional arrays of spike times. If we were to compute weight changes for each possible presynaptic–postsynaptic spike pair, then computational complexity drastically increases with the increase of simulation time and activity of adjacent neurons. A possible solution would be to compute weight changes only for some spikes that are closest in time. Calculating weight changes can be performed in two ways: one favourable to CPU and one to GPU architecture. In general, CPUs have higher clock rates and a lesser number of cores than GPUs. This causes them to be more efficient at executing loops; thus, the way favourable to CPU would be to conduct N “for” loops that have M iterations, where N is the number of postsynaptic spikes and M is the number of presynaptic spikes. This approach is computationally heavy but does not need a lot of memory. A better approach for GPUs, on the other hand, would be to create two MxN arrays from spike history arrays by copying one array to the size of the other across an additional axis. GPUs have a lot of cores and they allow for massive parallelization of operation. Due to this, computing the learning window on two arrays is efficient but can quickly drain available video random access memory (VRAM) of the GPU. On GPUs usually there is no swapping mechanism that can save memory to disc for later use in case of overload; thus, the amount of available VRAM is a hard constraint to machine learning practitioners.

Online implementation is a bit different than the standard one, because in this variant weight changes are calculated during network’s forward pass and not after. In this approach, instead of storing spike history, two variables are maintained: presynaptic and postsynaptic trace. The former is increased whenever a presynaptic spike arrives and the latter is increased whenever a postsynaptic spike arrives. Both decay exponentially in the absence of spikes. The weight update is calculated on each postsynaptic spike [26]. STDP learning windows are also adjusted to this new mechanism. An example is shown below,

(28) $$\Delta w=\eta \left(x_{pre}-x_{tar}\right){\left(w_{max}-w\right)}^{\mu },$$

where $x_{pre}$ and $x_{tar}$ are presynaptic and postsynaptic traces, η is the learning rate, *w* is the weight, $w_{max}$ is the maximum weight allowed, and μ determines dependence on the previous weight and max weight during weight update. There are many trace-based STDP learning windows [26]. Online implementation is a lot more efficient than the standard one on both CPU and GPU, because it minimizes the overhead of weight update calculations. It also achieves comparable results on metrics to the standard version [26,35]. For GPU architectures, it is even more beneficial, because now there is no need to compute weight updates by using MxN-shaped arrays. However, concurrency capability is not wasted in this case, as it can be used to parallelize the querying network on whole batches and computing updates per layers of neurons, instead of single neurons.

### 4.2. Backpropagation

Backpropagation-based SNN learning approaches are usually implemented in deep learning frameworks, such as tensorflow and PyTorch. These frameworks use various techniques of autodifferentiation (auto-diff for short) to compute the derivatives during the backpropagation algorithm. These techniques do not evaluate derivatives as expressions but evaluate derivatives in numeric form. This is done by accumulating values during code execution (forward pass of the network). Specifically in backpropagation, standard computation is augmented with derivative computation and through the chain rule the gradients are computed [66]. However, when it comes to SNNs, the full derivative term is incomputable; thus, this derivative is substituted somehow, as was mentioned in Section 3.2.

Backpropagation training is performed in batches of data, in which the batch size greatly influences the stability of the training [27]. In general, GPUs vastly outperform CPUs in that regard, especially on higher batch sizes, as the forward passes for all examples in the batch are computed concurrently. Typical batch sizes used for most problems are 4, 8, 16, 32, and 64. With the increase of batch size, VRAM requirements also increase, and this makes the available memory of the GPU a hard constraint during training. This parallelization also takes place in the computation of derivatives.

Backpropagation in SNNs can be realized by calculating the derivative only on spike times (Spikeprop), all of the time steps (Superspike, SLAYER), or only by using spike times themselves (SSTDP). All of these variants differ in terms of computational complexity. The latter category is the fastest, as the computation per layer is largely singular and can be parallelized across all neurons in a layer. In the case of SSTDP, there is only one spike per neuron, so the paradigm of singular equation can be fulfilled. As for the rest of the methods, the derivatives for certain time steps may need to be calculated separately. For methods that do not take into consideration temporal dependency between subsequent spikes, such as Spikeprop and Superspike, it is not the case, but for SLAYER the calculation needs to be conducted separately for each time step, as the current time step is expressed as a function of previous time steps [56]. This behaviour is very similar to the backpropagation through time (BPTT) algorithm, which is used to train recurrent neural networks [67].

### 4.3. ANN-SNN Conversion

For the ANN-SNN conversion, computational complexity of the training is largely similar to the computational complexity of training regular ANNs, as the overhead that comes from calculation of output SNN parameters is relatively small. These calculations are only performed one time per SNN, after the training of the source ANN.

### 4.4. Computational Complexity Comparison

In order to compare all types of learning approaches, we decided to measure batch processing times on CPU and GPU, as well as peak memory usage for GPU. For each group of learning approaches we picked one algorithm to represent it. For STDP, we have used a trace-based implementation, using an update rule from Equation (28). For backpropagation, we have chosen SSTDP to represent this category as it is more efficient that most backpropagation-based SNN learning algorithms, while maintaining good results on metrics [42]. Lastly, for ANN-SNN conversion, we have measured ANN training of identical structure to the compared SNNs from the other two algorithms. Because the output SNN parameter calculation time is small, we decided not to include it in the calculations. For the benchmarking dataset, we have chosen MNiST.

SNN architectures for STDP and SSTDP were identical. Networks consisted of two layers of 600 and 10 spike-once I&F neurons. Inputs followed temporal encoding, as is described in Section 2.3.2 and simulation steps were set to 32 for both SNNs. We’ve chosen spike-once I&F neurons and temporal encoding to maintain a high degree of comparability between STDP and SSTDP, as the latter demands these two properties out of the network architecture. ANN, representing ANN-SNN conversion, consisted of two dense layers of 600 neurons with ReLU activation function and 10 neurons with softmax activation function. The Adam optimizer with categorical cross-entropy loss was used for training purposes.

All implementations were performed by using the Tensorflow framework. The GPU used was Nvidia GeForce RTX 3080 and the CPU used was Intel Core i7-12700K. Each measurement was repeated 30 times, and the final result was an average of all repetitions. An important thing to note is that Tensorflow follows lazy initialization of its software components. This means that some memory allocations are performed at the time of the first graph query, and it causes the time to query the first batch to be much longer than the following batches. This occurrence is commonly referred to as “graph warm-up”. In our calculations we have skipped all first-batch processing times to avoid incorporating this overhead into our analysis.

**Figure 2:**
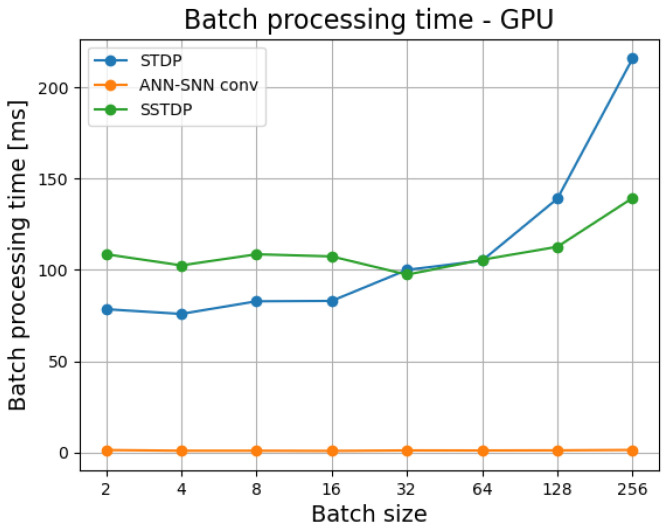
Batch processing times for different batch sizes on GPU.

Figure 2 presents GPU batch processing times for different batch sizes for all algorithms under comparison. Both SNNs, due to the necessity of conducting simulation over time steps, were vastly outperformed by an ANN, for which the batch processing time on all studied batch sizes was roughly equal to 1–2 ms. Interestingly, up until the batch size of 32, STDP outperforms SSTDP in terms of processing time, but after that mark the roles are reversed. This may be due to the necessity of maintaining a large array of trace variables for high number of neurons during STDP.

**Figure 3:**
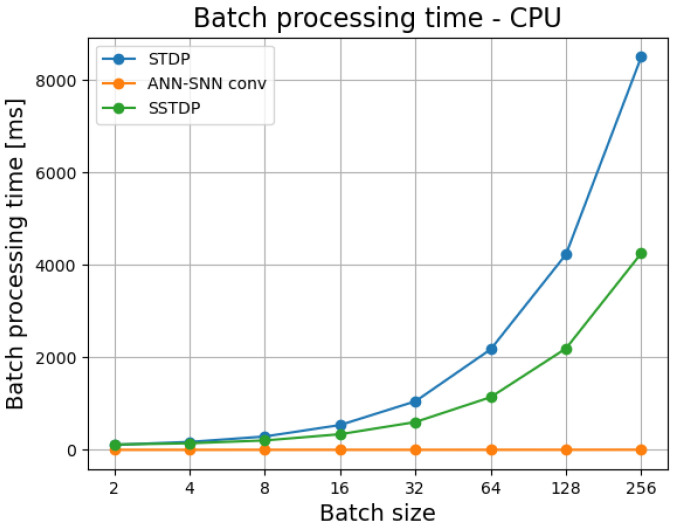
Batch processing times for different batch sizes on CPU.

Figure 3 presents CPU batch processing times for different batch sizes for all algorithms under comparison. Similar to the GPU case, ANN outperformed both SNNs. Batch processing times for the ANN on CPU equal to 2–4 ms. The differences between processing times on GPU and CPU for the ANN are minimal, most likely due to the small size of the network. These differences, however, would be much higher if the network’s architecture was larger. On CPU, SSTDP and STDP have almost equal performance up to the batch size of 8 and after that the former outperforms the latter. Overall, batch processing times for both SNNs on CPU are much longer than processing times on GPU (around 20–30 times longer).

**Figure 4:**
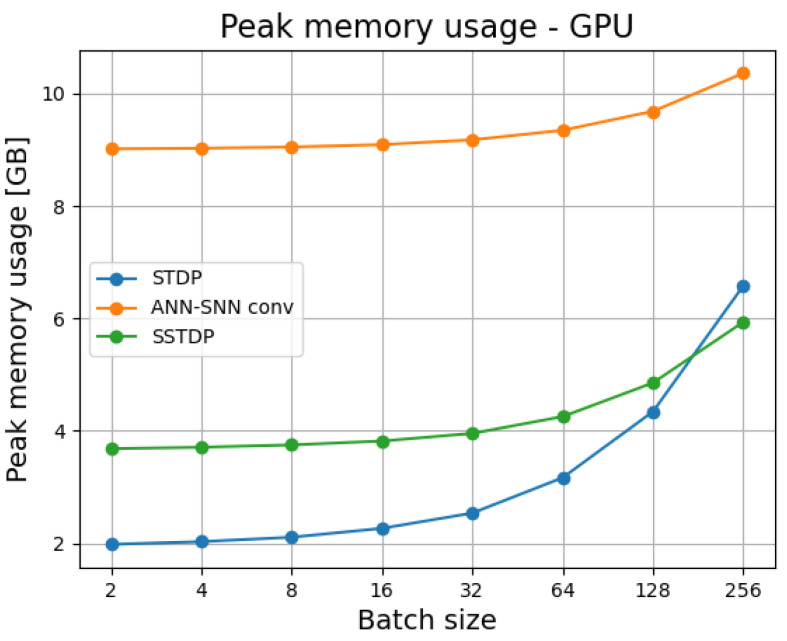
Batch processing times for different batch sizes on GPU.

Figure 4 presents GPU memory usage for different batch sizes for all algorithms under comparison. The ANN had the biggest memory usage. This may be due to the individual factors of ready-to-use backpropagation implementation in Tensorflow, as SSTDP and STDP implementations were custom-made for our analyses. For most batch sizes tested STDP requires less memory than SSTDP. This can, however, be just a trait of our implementation.

Although the results were obtained only on one dataset, their importance can be carried over to other datasets as well, due to the fact that the input data format for feedforward SNNs is universal (vector with numerical values). With this in mind, we can state that these results may slightly deviate for convolutional SNNs, as the data in them is represented as four-dimensional arrays. The general trend of ANN training being much faster will be maintained though, as the necessity of querying SNNs over multiple time steps is universal across all SNN architectures.

One other factor must be mentioned—the choice of the software framework. In this case we believe that it does influence the results obtained, as the simulations were performed in Tensorflow’s graph mode. Graph mode is the preferred method of code execution in Tensorflow, which forms graphs from operations, optimizes them, and runs them on designated hardware. It provides the optimal way of operation execution for the given hardware. For CPUs, it means the use of looping and vectorization. For GPUs, it means the parallelization of operations (operations performed on all elements of arrays simultaneously). CPU’s vectorization is a hardware feature that allows us to parallelize operations to a small extent. This ability, however, is vastly inferior to GPU’s capabilities for parallelization. According to our knowledge, these results will also hold true for PyTorch framework, as the algorithms used for optimization there are similar to Tensorflow’s.

Table 2 summarizes all elements that add to computational complexity for every learning approach type mentioned in this paper. For STDP, we only take into account the best scenario—online implementation, as the speed of training in standard implementation is heavily dependent on hardware resources, such as VRAM. Although the ANN-SNN conversion produces the mostly costly SNN networks due to the rate encoding prevalence, it is least computationally complex on standard computer hardware (CPU, GPU).

## 5. Conclusions

In this paper we have presented an overview of spiking neural network fundamentals, their learning algorithms, and corresponding computational complexities of those algorithms for synchronous digital computer hardware. SNNs are a trending topic among machine learning researchers, as the energy cost reduction possible to achieve from them is significant and may enable further progress in the broad field of neural networks, where hardware and its maintenance costs are often the limiting factors. As of now, SNNs are ill-suited to run on standard computer hardware due to the necessity of conducting simulation over several time steps, but because of the inavailability of neuromoprhic hardware and its limited simulation capabilities, as of the time of writing, they have to be studied on such hardware. Perhaps in the future, with the spread of neuromorphic hardware platforms’ availability and their standardization, the situation will change.

## Figures and Tables

**Table 1:** Highest accuracy on MNiST per approach.

	MNiST	CIFAR-10
STDP	97.20% [64]	-
Backpropagation	99.72% [57]	93.50% [57]
ANN-SNN conversion	99.44% [57]	93.63% [65]

**Table 2:** SNN learning approaches complexity comparison.

	Online STDP	Backpropagation	ANN-SNN Conversion
SNN forward pass	√	√	-
ANN forwads pass	-	-	√
trace-based weight update	√	-	-
Backpropagation	-	√	√
param calculation	-	-	√

## Data Availability

No new data were created or analyzed in this study. Data sharing is not applicable to this article.

## Appendix A. Variable Names

The following variable designations are used in the equations:

C	number of classess	Equation (22)
$C_{m}$	membrane capacitance	Equations (1) and (2)
V, v	previous membrane potential	Equations (1)–(5) and (7)
$v^{l}$	neuron’s potential after firing	Equation (27)
u	recovery variable	Equations (3)–(5)
I	total current from synapses	Equations (1)–(3) and (9)
$g_{L}$	leak conductance	Equations (1) and (2)
g	synapse conductance	Equations (8)–(10) and (17)
$E_{K}$	potassium reversal potentials	Equation (6)
$E_{L}$	leak reversal potential	Equations (1), (2) and (6)
$E_{Na}$	sodium reversal potentials	Equation (6)
$E_{eq}$	equilibrium synapse potential	Equation (9)
a, b, c, d	constants	Equations (3)–(5)
$I_{ion}$	total current through the membrane	Equations (6) and (7)
$I_{syn}$	synaptic input current	Equation (7)
$G_{K}$	potassium conductances per unit area	Equation (6)
$G_{Na}$	sodium conductances per unit area	Equation (6)
$G_{L}$	leak conductance per unit area	Equation (6)
m, h, n	constants	Equation (6)
n	number of output spikes	Equation (17)
$v_{m}$	membrane potential	Equations (6) and (16)
$v_{sp}$	spike voltage level	Equations (8) and (9)
$\tau_{g_{e}}$	time constant of an excitatory postsynaptic potential	Equation (10)
Δ	learning window	Equations (11) and (12)
$A_{+},A_{-}$	scaling factors for potentiation and depression	Equations (11) and (12)
$t_{pre},t_{post}$	presynaptic and postsynaptic spike times	Equations (11) and (12)
$\tau_{+},\tau_{-}$	time constants of synapse potentiation and depression	Equations (11) and (12)
$\eta_{+},\eta_{-}$	potentiation and depression learning rates	Equation (13)
w	weight value	Equation (13)
W	weight matrix	Equation (25) and (27)
E	mean squares error	Equations (14) and (17)
$t_{j}$	actual spike time	Equations (14) and (17)
$t_{j}^{d}$	desired spike time	Equation (14)
L	loss function	Equations (15), (20) and (22)
α	normalised smooth temporal convolution kernel	Equation (15)
*s*	output spike train	Equation (15)
$s^{\prime }$	target spike train	Equation (15)
σ	sigmoid function	Equations (16), (23) and (24)
T	expected firing time	Equations (17) and (22)
$T_{mean}$	average firing time	Equation (17)
ϵ	spike response kernel	Equations (19) and (21)
μ	constant	Equation (19)
o	output tensor	Equation (22)
$a_{l}$	output of layers	Equation (25)
h	ReLU activation function	Equations (25) and (26)
ϕ	avarage postsynaptic potential	Equation (27)

## Abbreviations

The following abbreviations are used in this manuscript:

ANN	Artificial Neural Network
BPTT	Back Propagation Through Time
CNN	Convolutional Neural Network
CPU	Central Processing Unit
DVS	Dynamic Vision Sensors
FPGA	Field Programmable Gate Arrays
GPU	Graphics Processing Unit
IF	Integrate and Fire
LIF	Leaky Integrate and Fire
NEF	Neural Engineering Framework
ReLU	Rectified Linear Unit
R-STDP	Reward Modulated Spike Timing Dependent Plasticity
SLAM	Simultaneous Localization and Mapping
SNN	Spiking Neural Network
SSTDP	Supervised Spike Timing Dependent Plasticity
STDP	Spike Timing-Dependent-Plasticity
TPU	Tensor Processing Unit
VPU	Vision Processing Units
VRAM	Video Random Access Memory
WTA	Winner Takes All

## References

[1] Wang C.-Y., Bochkovskiy A., Liao H.-Y.M. (2022). YOLOv7: Trainable Bag-of-Freebies Sets New State-of-the-Art for Real-Time Object Detectors. arXiv.

[2] Ronneberger O., Fischer P., Brox T. (2015). U-Net: Convolutional networks for biomedical image segmentation. Medical Image Computing and Computer-Assisted Intervention (MICCAI).

[3] Brown T., Mann B., Ryder N., Subbiah M., Kaplan J.D., Dhariwal P., Neelakantan A., Shyam P., Sastry G., Askell A., Larochelle H., Ranzato M., Hadsell R., Balcan M.F., Lin H. (2020). Language Models Are Few-Shot Learners. Advances in Neural Information Processing Systems.

[4] LeCun Y., Bottou L., Bengio Y., Ha P. (1998). Gradient-Based Learning Applied to Document Recognition. Proc. IEEE.

[5] Krizhevsky A. Learning Multiple Layers of Features from Tiny Images. https://www.cs.toronto.edu/kriz/learning-features-2009-TR.pdf.

[6] Zhou S., Chen Y., Li X., Sanyal A. (2020). Deep SCNN-Based Real-Time Object Detection for Self-Driving Vehicles Using LiDAR Temporal Data. IEEE Access.

[7] Patel K., Hunsberger E., Batir S., Eliasmith C. (2021). A Spiking Neural Network for Image Segmentation. arXiv.

[8] Shalumov A., Halaly R., Tsur E.E. (2021). LiDAR-driven spiking neural network for collision avoidance in autonomous driving. Bioinspiration Biomimetics.

[9] Baby S.A., Vinod B., Chinni C., Mitra K. (2017). Dynamic vision sensors for human activity recognition. Proceedings of the 2017 4th IAPR Asian Conference on Pattern Recognition (ACPR).

[10] Orchard G., Jayawant A., Cohen G.K., Thakor N. (2015). Converting Static Image Datasets to Spiking Neuromorphic Datasets Using Saccades. Front. Neurosci..

[11] Li H., Liu H., Ji X., Li G., Shi L. (2017). CIFAR10-DVS: An Event-Stream Dataset for Object Classification. Front. Neurosci..

[12] Han B., Roy K., Vedaldi A., Bischof H., Brox T., Frahm J.M. (2020). Deep spiking neural network: Energy efficiency through time based coding. Computer Vision—ECCV 2020.

[13] Makarov V.A., Lobov S.A., Shchanikov S., Mikhaylov A., Kazantsev V.B. (2022). Toward Reflective Spiking Neural Networks Exploiting Memristive Devices. Front. Comput. Neurosci..

[14] Lobov S.A., Zharinov A.I., Makarov V.A., Kazantsev V.B. (2021). Spatial Memory in a Spiking Neural Network with Robot Embodiment. Sensors.

[15] Mo L., Wang G., Long E., Zhuo M. (2022). ALSA: Associative Learning Based Supervised Learning Algorithm for SNN. Front. Neurosci..

[16] Tang G., Shah A., Michmizos K.P. Spiking Neural Network on Neuromorphic Hardware for Energy-Efficient Unidimensional SLAM. Proceedings of the IEEE/RSJ International Conference on Intelligent Robots and Systems (IROS).

[17] Juarez-Lora A., Ponce-Ponce V.H., Sossa H., Rubio-Espino E. (2022). R-STDP Spiking Neural Network Architecture for Motion Control on a Changing Friction Joint Robotic Arm. Front. Neurorobot..

[18] Sandamirskaya Y., Kaboli M., Conradt J., Celikel T. (2022). Neuromorphic computing hardware and neural architectures for robotics. Sci. Robot..

[19] DeWolf T. (2021). Spiking neural networks take control. Sci. Robot..

[20] Furber S., Yan Y., Stewart T., Choo X., Vogginger B., Partzsch J., Hoeppner S., Kelber F., Eliasmith C., Mayr C. (2021). Comparing Loihi with a SpiNNaker 2 prototype on low-latency keyword spotting and adaptive robotic control. Neuromorphic Comput. Eng..

[21] DeWolf T., Stewart T.C., Slotine J.J., Eliasmith C. (2016). A spiking neural model of adaptive arm control. Proc. Biol Sci..

[22] Falanga D., Kleber K., Scaramuzza D. (2020). Dynamic obstacle avoidance for quadrotors with event cameras. Sci. Robot..

[23] DeWolf T., Jaworski P., Eliasmith C. (2020). Nengo and Low-Power AI Hardware for Robust, Embedded Neurorobotics. Front. Neurorobot..

[24] Ivanov D., Chezhegov A., Grunin A., Kiselev M., Larionov D. (2022). Neuromorphic Artificial Intelligence Systems. arXiv.

[25] Quian Quiroga R., Kreiman G. (2010). Measuring Sparseness in the Brain: Comment on Bowers (2009). Psychol. Rev..

[26] Diehl P.U., Cook M. (2015). Unsupervised Learning of Digit Recognition Using Spike-Timing-Dependent Plasticity. Front. Comput. Neurosci..

[27] Rumelhart D., Hinton G., Williams R. (1986). Learning representations by back-propagating errors. Nature.

[28] Eliasmith C., Anderson C.H. (2003). Neural Engineering: Computation, Representation, and Dynamics in Neurobiological Systems.

[29] Tsur E.E. (2021). Neuromorphic Engineering: The Scientist’s, Algorithm Designer’s, and Computer Architect’s Perspectives on Brain-Inspired Computing.

[30] Voelker A.R., Eliasmith C. (2020). Programming neuromorphics using the neural engineering framework. Handbook of Neuroengineering.

[31] Akopyan F., Sawada J., Cassidy A., Alvarez-Icaza R., Arthur J., Merolla P., Imam N., Nakamura Y., Datta P., Nam G.-J. (2015). TrueNorth: Design and Tool Flow of a 65 MW 1 Million Neuron Programmable Neurosynaptic Chip. IEEE Trans. Comput.-Aided Des. Integr. Circuits Syst..

[32] Davies M., Srinivasa N., Lin T.-H., Chinya G., Cao Y., Choday S.H., Dimou G., Joshi P., Imam N., Jain S. (2018). Loihi: A Neuromorphic Manycore Processor with On-Chip Learning. IEEE Micro.

[33] Höppner S., Yan Y., Dixius A., Scholze S., Partzsch J., Stolba M., Kelber F., Vogginger B., Neumärker F., Ellguth G. (2022). The SpiNNaker 2 Processing Element Architecture for Hybrid Digital Neuromorphic Computing. arXiv.

[34] Moreira O., Yousefzadeh A., Chersi F., Kapoor A., Zwartenkot R.-J., Qiao P., Cinserin G., Khoei M.A., Lindwer M., Tapson J. (2020). NeuronFlow: A Hybrid Neuromorphic—Dataflow Processor Architecture for AI Workloads. Proceedings of the 2020 2nd IEEE International Conference on Artificial Intelligence Circuits and Systems (AICAS).

[35] Yamazaki K., Vo-Ho V.-K., Bulsara D., Le N. (2022). Spiking Neural Networks and Their Applications: A Review. Brain Sci..

[36] Szczęsny S., Huderek D., Przyborowski Ł. (2021). Spiking Neural Network with Linear Computational Complexity for Waveform Analysis in Amperometry. Sensors.

[37] Szczęsny S., Kropidłowski M., Naumowicz M. (2020). 0.50-V ultra-low-power ΣΔ modulator for sub-nA signal sensing in amperometry. IEEE Sens. J..

[38] Szczęsny S., Huderek D., Przyborowski Ł. (2023). Explainable spiking neural network for real time feature classification. J. Exp. Theor. Artif. Intell..

[39] Van Pottelbergh T., Drion G., Sepulchre R. (2018). Robust Modulation of Integrate-and-Fire Models. Neural Comput..

[40] Dutta S., Kumar V., Shukla A., Mohapatra N.R., Ganguly U. (2017). Leaky Integrate and Fire Neuron by Charge-Discharge Dynamics in Floating-Body MOSFET. Sci. Rep..

[41] Izhikevich E.M. (2003). Simple Model of Spiking Neurons. IEEE Trans. Neural Netw..

[42] Liu F., Zhao W., Chen Y., Wang Z., Yang T., Jiang L. (2021). SSTDP: Supervised Spike Timing Dependent Plasticity for Efficient Spiking Neural Network Training. Front. Neurosci..

[43] Sengupta A., Ye Y., Wang R., Liu C., Roy K. (2019). Going Deeper in Spiking Neural Networks: VGG and Residual Architectures. Front. Neurosci..

[44] Pfeiffer M., Pfeil T. (2018). Deep Learning With Spiking Neurons: Opportunities and Challenges. Front. Neurosci..

[45] Markram H., Gerstner W., Sjöström P.J. (2012). Spike-Timing-Dependent Plasticity: A Comprehensive Overview. Front. Synaptic Neurosci..

[46] Song S., Miller K., Abbott L. (2000). Competitive Hebbian learning through spike-timing-dependent synaptic plasticity. Nat. Neurosci..

[47] Izhikevich E. (2007). Solving the Distal Reward Problem through Linkage of STDP and Dopamine Signaling. Cereb. Cortex.

[48] Burbank K.S. (2015). Mirrored STDP Implements Autoencoder Learning in a Network of Spiking Neurons. PLoS Comput. Biol..

[49] Masquelier T., Thorpe S.J. (2007). Unsupervised Learning of Visual Features through Spike Timing Dependent Plasticity. PLoS Comput. Biol..

[50] Vigneron A., Martinet J. (2020). A Critical Survey of STDP in Spiking Neural Networks for Pattern Recognition. Proceedings of the 2020 International Joint Conference on Neural Networks (IJCNN).

[51] Zhong X., Pan H. (2022). A Spike Neural Network Model for Lateral Suppression of Spike-Timing-Dependent Plasticity with Adaptive Threshold. Appl. Sci..

[52] Yousefzadeh A., Stromatias E., Soto M., Serrano-Gotarredona T., Linares-Barranco B. (2018). On Practical Issues for Stochastic STDP Hardware With 1-Bit Synaptic Weights. Front. Neurosci..

[53] Bohte S., Kok J., Poutré J. SpikeProp: Backpropagation for Networks of Spiking Neurons. Proceedings of the 8th European Symposium on Artificial Neural Networks, ESANN 2000.

[54] Zenke F., Ganguli S. (2018). SuperSpike: Supervised Learning in Multi-Layer Spiking Neural Networks. Neural Comput..

[55] O’Connor P., Welling M. (2016). Deep Spiking Networks. arXiv.

[56] Shrestha S.B., Orchard G., Bengio S., Wallach H., Larochelle H., Grauman K., Cesa-Bianchi N., Garnett R. (2018). SLAYER: Spike Layer Error Reassignment in Time. Advances in Neural Information Processing Systems.

[57] Fang W., Yu Z., Chen Y., Masquelier T., Huang T., Tian Y. (2021). Incorporating Learnable Membrane Time Constant to Enhance Learning of Spiking Neural Networks. Proceedings of the 2021 IEEE/CVF International Conference on Computer Vision (ICCV).

[58] Esser S.K., Appuswamy R., Merolla P., Arthur J.V., Modha D.S. (2015). Backpropagation for Energy-Efficient Neuromorphic Computing.

[59] Diehl P.U., Zarrella G., Cassidy A., Pedroni B.U., Neftci E. Conversion of Artificial Recurrent Neural Networks to Spiking Neural Networks for Low-Power Neuromorphic Hardware. Proceedings of the 2016 IEEE International Conference on Rebooting Computing (ICRC).

[60] Stöckl C., Maass W. (2020). Recognizing Images with at Most One Spike per Neuron. arXiv.

[61] Bu T., Fang W., Ding J., Dai P., Yu Z., Huang T. (2022). Optimal Ann-Snn Conversion for High- Accuracy and Ultra-Low-Latency Spiking Neural Networks.

[62] Cao Y., Chen Y., Khosla D. (2015). Spiking deep convolutional neural networks for energy-efficient object recognition. Int. J. Comput. Vis..

[63] Rueckauer B., Lungu I.-A., Hu Y., Pfeiffer M., Liu S.-C. (2017). Conversion of Continuous-Valued Deep Networks to Efficient Event-Driven Networks for Image Classification. Front. Neurosci..

[64] Mozafari M., Ganjtabesh M., Nowzari-Dalini A., Thorpe S.J., Masquelier T. (2019). Bio-Inspired Digit Recognition Using Reward-Modulated Spike-Timing-Dependent Plasticity in Deep Convolutional Networks. Pattern Recognit..

[65] Han B., Srinivasan G., Roy K. RMP-SNN: Residual Membrane Potential Neuron for Enabling Deeper High-Accuracy and Low-Latency Spiking Neural Network. Proceedings of the IEEE Conference on Computer Vision and Pattern Recognition.

[66] Baydin A., Pearlmutter B., Radul A., Siskind J. (2018). Automatic Differentiation in Machine Learning: A Survey. J. Mach. Learn. Res..

[67] Werbos P.J. (1990). Backpropagation through time: What it does and how to do it. Proc. IEEE.

